# Molecular and Functional Platelet Abnormalities in Myeloproliferative Neoplasms

**DOI:** 10.3390/cells15060555

**Published:** 2026-03-19

**Authors:** Ann X. Wang, Belinda B. Guo, Matthew D. Linden

**Affiliations:** School of Biomedical Science, University of Western Australia, Perth, WA 6009, Australia

**Keywords:** platelets, myeloproliferative neoplasms, myelofibrosis, megakaryocytes, platelet function, platelet transcriptome, platelet proteome, thrombosis, hemostasis, biomarker

## Abstract

Blood platelets are derived from megakaryocytes with functions extending beyond hemostasis to inflammation, immunity, and cancer. Myeloproliferative neoplasms (MPNs) are clonal stem cell disorders driven by somatic mutations affecting JAK-STAT signaling, leading to excessive myeloid proliferation. Thrombosis affects approximately one-fifth of patients at diagnosis and remains elevated throughout the disease course, while the paradoxical coexistence of bleeding further complicates clinical management. In addition, MPNs may progress to advanced disease stages, including bone marrow fibrosis and transformation to acute myeloid leukemia, leading to ineffective hematopoiesis, worsening symptom burden, and poor clinical outcomes. This review outlines how peripherally circulating platelets provide a unique window into MPN pathophysiology, with emphasis on their functional and molecular abnormalities. We summarize current understanding of platelet-mediated hemostatic imbalance across MPN subtypes. We discuss the potential of platelet transcriptomics and proteomics to reveal disease-specific signatures. We further highlight emerging platelet-associated candidates with potential utility as dynamic biomarkers for both the pathological marrow niche and thrombotic and bleeding risk. Together, these insights underscore the potential of platelet-based approaches to complement existing diagnostic and prognostic strategies in MPNs.

## 1. Introduction

Platelets are best known for their essential role in maintaining vascular integrity and mediating hemostasis [1]. Beyond these classical functions, platelets have been extensively studied in solid tumors for their contributions to immune evasion, angiogenesis, and metastasis [1]. More recently, growing evidence has extended their involvement into hematological malignancies.

Myeloproliferative neoplasms (MPNs) are clonal hematopoietic stem cell disorders characterized by the excessive production of mature myeloid cells, driven predominantly by mutations in *JAK2*, *CALR*, or *MPL* that constitutively activate the JAK-STAT pathway [2]. The classical MPN subtypes include polycythemia vera (PV), essential thrombocythemia (ET), pre-fibrotic myelofibrosis, and primary myelofibrosis (PMF) [3]. Across all MPNs, hemostatic complications affect approximately 20% of patients and represent a major cause of early morbidity and mortality [4,5,6,7]. Progression to advanced disease stages, including myelofibrosis (MF) and acute myeloid leukemia (AML), occurs in roughly 5–20% depending on disease subtype and duration and is associated with increased disease burden and reduced survival [4,8,9].

As a consequence of oncogenic signaling-driven myeloproliferation, platelet overproduction is a defining feature of ET, while PV is characterized primarily by erythrocytosis with concomitant megakaryocytic hyperplasia and increased platelet output [10]. MF is marked by bone marrow (BM) architectural disruption and ineffective hematopoiesis, in which platelet counts are more heterogeneous and may decline with disease progression, often accompanied by abnormal morphological features such as altered size and granularity [10].

Beyond quantitative platelet abnormalities in MPNs, emerging studies have described a paradoxical platelet phenotype characterized by baseline activation, altered adhesive and signaling properties, and impaired hemostatic responsiveness [11,12]. As the management of major hemostatic complications in MPNs remains a persistent clinical challenge, platelet functional abnormalities warrant greater consideration in current clinical risk-stratification models and treatment guidelines.

During thrombopoiesis, the platelet molecular repertoire is predominantly inherited from their parent megakaryocytes, which in MPNs are clonal and exhibit aberrant differentiation, cytokine secretion, and niche remodeling that drive marrow fibrosis and systemic inflammation [13,14,15]. This inherited profile might be further modulated in circulation by the systemic inflammatory and prothrombotic milieu in MPNs as platelets are capable of activation-dependent signal transduction and post-transcriptional reprogramming [11,16]. As a result, circulating platelets integrate intrinsic clonal alterations with extrinsic disease-associated signals, positioning them as dynamic reporters of disease state. Given their short lifespan, platelet molecular profiles are inherently dynamic, and longitudinal platelet profiling may therefore offer a means to monitor disease evolution and therapeutic response [17].

This review synthesizes current knowledge on platelet pathology in MPNs. We discuss insights from platelet transcriptomic and proteomic profiling, highlight emerging candidate biomarkers and therapeutic targets, and examine how platelet dysfunction contributes to both thrombotic and hemorrhagic risk. By integrating these perspectives, a systematic understanding of platelet biology from molecular imprint to functional output could bridge critical gaps in MPN management, offering novel avenues for diagnostic refinement, dynamic risk assessment, and the development of targeted therapies.

## 2. Platelet Biology

Platelets are small, anucleate cell fragments with a characteristic discoid shape, typically ranging from 1 to 3 µm in diameter [18]. Under normal physiological conditions, platelets are essential for maintaining vascular integrity by initiating clot formation and contributing to wound healing, angiogenesis, inflammation, and innate immunity [18].

Platelets are produced by megakaryocytes, large, rare BM cells that also help modulate the BM niche [18]. Megakaryocytes arise from CD34^+^ hematopoietic stem cells through myeloid progenitors under the influence of thrombopoietin (TPO), which signals via its receptor MPL [19]. Engagement of the TPO/MPL axis activates downstream pathways including JAK2-STAT3/5 and mitogen-activated protein kinase (MAPK), driving megakaryocyte maturation and thrombopoiesis [18,19]. During differentiation, megakaryocytes undergo endomitosis, a modified cell cycle characterized by DNA replication without cytokinesis, resulting in highly polyploid cells with marked cytoplasmic expansion [20]. This process is accompanied by RNA synthesis and storage, generating a diverse RNA repertoire [21]. When megakaryocytes begin forming the long and branching extensions, selective RNA molecules and proteins are transported along these structures and incorporated into platelets [21].

As platelets circulate, their molecular profiles are dynamic and can be highly informative, shifting with changes in disease burden and in response to therapy [22]. In solid tumors, circulating platelets acquire tumor-associated signatures through sequestration of tumor- or stroma-derived exosomes and microvesicles carrying RNA and protein cargo, as well as receptor-mediated endocytosis of soluble circulating factors originating from the tumor microenvironment [23]. RNA profiles in these tumor-educated platelets can differentiate cancer patients from healthy individuals, help classify tumor types, and reflect active oncogenic pathways, underscoring their potential as minimally invasive biomarkers for cancer detection and monitoring [24].

## 3. Myeloproliferative Neoplasms

**Figure 1:**
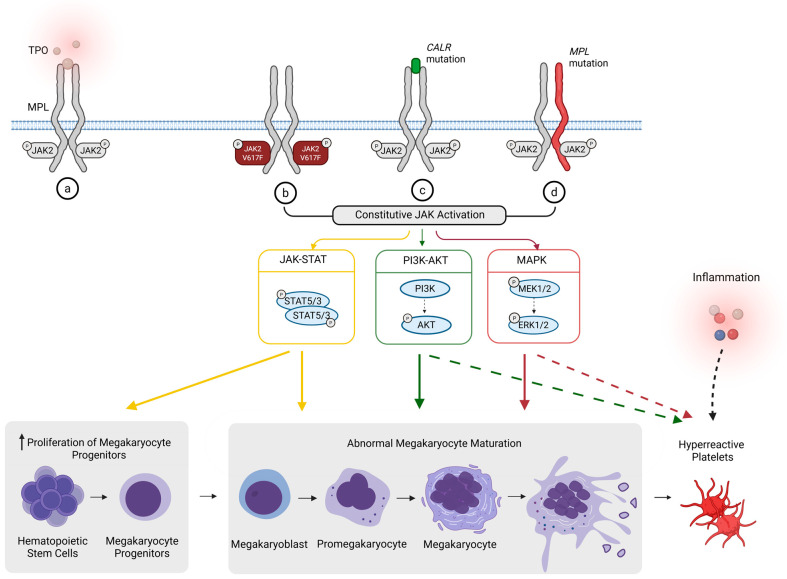
*JAK2*, *CALR*, and *MPL* mutations dysregulate megakaryopoiesis and platelet function. (**a**) While physiologic TPO binding induces MPL dimerization and transient JAK activation, in MPNs; (**b**) *JAK2*^V617F^ is a gain-of-function JAK2 point mutation that amplifies signaling from MPL-tethered JAK2, reducing TPO dependence, (**c**) *CALR* mutations generate mutant CALR that aberrantly engages MPL and drives TPO-independent receptor activation, and (**d**) *MPL* mutations alter the TPO-receptor (MPL) to an active conformation, causing ligand-independent signal transduction. In all cases, activated JAK2 phosphorylates STAT5/3 (yellow), promoting STAT dimerization and nuclear translocation to drive transcriptional programs that support megakaryocyte progenitor expansion and aberrant megakaryopoiesis. Constitutive MPL-JAK2 signaling also activates PI3K and downstream Akt (green). Phosphorylated Akt modulates nuclear transcriptional regulators to abnormal megakaryocyte maturation process and abnormal platelet production. In parallel, MPL-JAK2 engages MAPK signaling (red), with phosphorylation of MEK1/2 and downstream Extracellular signal-regulated kinase 1/2 (ERK1/2) activation. Phosphorylated ERK1/2 translocates to the nucleus to phosphorylate transcription factors that contribute to aberrant megakaryocyte maturation and disordered proplatelet formation. In platelets, upregulated PI3K-Akt and MAPK signaling pathways amplify platelet activation and contribute to a hyperreactive phenotype (green and red dashed arrows). Additionally, the platelet hyperreactive phenotype is also influenced by the inflammatory environment (black dashed arrow; Section 4.1).

Approximately 90% of MPNs are driven by somatic mutations in *JAK2*, *CALR*, or *MPL* [25]. The remaining 10%, which lack these mutations, are classified as triple-negative [25]. The somatic mutations converge on MPL-JAK2 signaling, resulting in persistent JAK2 kinase activity and downstream STAT/PI3K/MAPK pathway activation and preferentially promoting megakaryocytic differentiation and proliferation (Figure 1) [19,26,27,28]. The same constitutive signaling persists during megakaryocyte maturation by enhancing endomitosis and cytoplasmic expansion and dysmorphia, leading to hyperpolyploid megakaryocytes and abnormal clustering [10,26,29]. These abnormal megakaryocytes are a source of pro-inflammatory and pro-fibrotic cytokines (e.g., TGF-β, PDGF), contributing to the BM microenvironment remodeling and fibrosis seen in advanced disease [13,14].

Alteration of megakaryocytes dynamics impact on forming proplatelet extensions, leading to inefficient or disordered platelet shedding [30,31]. Therefore, abnormal megakaryocytes produce a high volume of qualitatively abnormal and dysfunctional platelets. Additionally, *JAK2*, *CALR*, or *MPL* mutations may prime platelets toward a hyperreactive phenotype. In platelets, Phosphoinositide 3-kinase-Akt (PI3K-Akt) acts as a key amplification pathway supporting integrin αIIbβ3 activation, secretion, and stable aggregation [32,33,34]. MAPK pathways can reinforce activation in an agonist-dependent manner, including regulation of thromboxane-linked feedback [35]. Sustained MPL-JAK2 signaling is therefore expected to magnify downstream PI3K-Akt and MAPK responses, contributing to exaggerated platelet reactivity and thrombus formation.

MPNs follow a chronic disease course with lifelong risks of thrombosis and developing MF or transformation into a terminal blast phase resembling AML [2,36]. Median survival following diagnosis of overt MF is less than six years, while transformation to AML is associated with an extremely poor prognosis, with reported median survival of approximately 3.6 months and no currently available curative therapies [36,37].

Diagnosis and risk stratification in MPNs rely on clinical features, laboratory parameters, BM histopathology, and molecular testing, yet these approaches have limitations for longitudinal monitoring and early detection of disease evolution [3,38]. BM biopsy remains the diagnostic gold standard but is invasive and poorly suited to repeated assessment, particularly in MF, where excessive reticulin deposition can limit the ability to obtain representative aspirates and contributes to sampling variability [39]. In chronic MPNs such as ET and PV, clinical management is largely directed toward mitigating thrombotic risk, yet bleeding risk is difficult to balance and complicates the use of aspirin and cytoreductive therapy [40]. Moreover, prospective tools for assessing fibrotic progression remain limited [38]. As a result, overt MF is often diagnosed at a stage marked by clonal complexity and sustained inflammatory signaling, which limits the effectiveness of available therapies [39]. Collectively, these challenges highlight the need for deeper biological insight into hemostatic complications shared across MPNs, more sensitive tools for early diagnosis and dynamic risk stratification, and the development of novel therapeutic targets capable of intercepting fibrotic progression before irreversible disease ensues.

## 4. Functional Platelet Dysregulation in MPNs

### 4.1. Platelet-Mediated Thrombotic Risk

Thrombosis constitutes a major and often earlier source of morbidity and mortality across all disease stages, particularly in ET and PV [41]. PV carries the greatest thrombotic burden, with a prevalence of approximately 30%, followed by ET at around 20%, whereas PMF is associated with a comparatively lower risk of roughly 10% [41]. Thrombotic events frequently involve both arterial and venous systems, including life-threatening complications such as stroke, myocardial infarction, and thrombosis at atypical sites, such as splanchnic vein [41].

Clinical assessment of thrombotic risk in MPNs relies on risk stratification models to guide decisions regarding antiplatelet therapy and cytoreduction [42]. In addition to conventional risk factors including age over 60 years and a prior history of thrombosis, cardiovascular risk and the presence of *JAK2*^V617F^ mutation are incorporated into the international prognostic score of thrombosis for ET (IPSET) [42]. Although platelets play a central role in hemostasis, platelet functional parameters are not currently considered in thrombosis risk assessment models for MPNs.

Accumulating evidence indicates that platelets in MPNs can display a pre-activated, hyper-reactive phenotype. A recent patient study reported evidence of in vivo platelet activation in MPN, including increased surface P-selectin exposure and elevated platelet–leukocyte aggregates, suggesting a primed or hyperreactive phenotype [43]. Using an integrated multi-omic approach, the same study linked this phenotype to enrichment of PI3K-Akt-mTOR pathway (a key platelet activation–metabolic axis) and mitochondrial energy programs (energy metabolism supporting activation) in MPN platelets, supporting a molecular basis for platelet hyperreactivity [43]. The altered platelet activation phenotype is further supported by elevated circulating levels of soluble CD40 ligand and P-selectin, reflecting platelet α-granule secretion and ongoing in vivo activation [12].

The factors driving the hyperreactive platelet phenotype in MPNs remain incompletely defined and continue to be actively investigated. However, the MPN inflammatory milieu appears to contribute directly to this phenotype, as exposure of healthy control platelets to plasma from patients with MPN induces an activated immunophenotype [44].

The observed hyperreactive platelets are hypothesized to increase thrombotic risk by promoting adhesive interactions with leukocytes, enhancing platelet–leukocyte aggregate formation, and amplifying thromboinflammatory signaling [45,46].

The JAK1/2 inhibitors ruxolitinib and baricitinib have been shown in vitro to reduce platelet adhesion to collagen, aggregation, and granule secretion [47]. They likely target a crosstalk between JAK2/STAT5 and the PI3K/Akt signaling pathways and seem to preferentially affect collagen-mediated platelet activation [47]. In parallel, recent clinical data from more than 2000 MPN patients reveals a 48% decrease in thromboembolic events under JAK1/2 inhibitor therapy [48]. Together, these findings provide a rationale to investigate whether JAK1/2 inhibition directly modifies the hyperreactive platelet phenotype in vivo, and to what extent this platelet modulation drives the observed reduction in clinical thrombotic risk.

### 4.2. Platelet Hypofunction and Bleeding Risk

Hemorrhagic complications, although less prevalent than thrombosis, represent an important and clinically relevant feature of MPNs and often coexist paradoxically with thrombotic risk [49]. Bleeding manifestations range from mild mucocutaneous hemorrhage to major gastrointestinal or intracranial hemorrhage [50,51].

Bleeding complications are frequently observed despite normal or elevated platelet counts and are particularly prevalent in ET and MF. A well-recognized contributor to bleeding in MPNs is acquired von Willebrand syndrome (AvWS), particularly in ET, where high shear stress and extreme thrombocytosis promote adsorption and proteolytic degradation of high-molecular-weight von Willebrand factor multimers [41,50,51,52,53,54]. As a result, platelets circulate in a milieu where their primary adhesive ligand is being depleted, which sets the stage for impaired primary hemostasis and bleeding.

Additionally, platelets in MPNs exhibit increased but dysfunctional mitochondrial content that impairs their energetic reserve and compromises sustained activation responses, potentially limiting stable thrombus formation and thereby contributing to bleeding complications in some patients [55].

Simultaneously, available studies of platelet dysfunction in MPNs describe a paradoxical phenotype in which platelets appear pre-activated in vivo yet show impaired aggregation in vitro [12,44], a pattern that has been suggested to be consistent with platelet exhaustion. In MPNs, continued exposure to the inflammatory milieu keeps platelets in a “pre-activated” or “primed” state, meaning platelets might have already undergone activation and granule release in vivo. This ‘exhausted’ phenotype may reduce their capacity to respond appropriately to subsequent hemostatic stimuli.

Unlike thrombosis, bleeding risk in MPNs is not captured by formal risk stratification models and is instead assessed based on clinical features such as extreme thrombocytosis, AvWS prior bleeding history, MF with thrombocytopenia, and exposure to antiplatelet or anticoagulant therapy [50,54]. Management therefore relies on individualized clinical judgement, often requiring cytoreductive therapy to correct extreme thrombocytosis, modification or temporary discontinuation of antithrombotic agents, and targeted evaluation for hemostatic defects such as AvWS [52,53].

### 4.3. Barriers to Clinical Translation of Platelet Function Testing

The limited integration of platelet functional assessment into clinical practice reflects several practical and methodological challenges. Robust prospective evidence linking specific platelet functional phenotypes to thrombotic outcomes in MPNs remains scarce, and existing studies are often heterogeneous in design and endpoints. In addition, platelet function assays are highly sensitive to pre-analytical variables, including sample handling and processing time, which complicates standardization and reproducibility. It is also difficult to isolate platelet dysfunction as a primary cause of clinical outcomes, as it often coexists with other conditions like cardiovascular disease, confounding interpretation. Consequently, the clinical impact of platelet function testing is likely underestimated, which in turn limits its implementation. Overcoming these barriers will require efforts to standardize methodology and prospectively link platelet phenotypes to clinical outcomes. Incorporation of dynamic platelet functional readouts into existing thrombotic risk frameworks could enable more personalized and proactive management of hemostatic complications in MPNs.

## 5. Platelet Molecular Abnormalities in MPNs

### 5.1. Platelet Transcriptome

As anucleate cells, platelets lack the capacity for de novo transcription [21]. Instead, the majority of their RNA content is thought to be inherited from megakaryocytes during platelet biogenesis [21]. Therefore, aberrant megakaryopoiesis in MPNs may establish disease-specific molecular imprint in circulating platelets. Upon release into the circulation, this baseline platelet transcriptomic state is further modified through activation-associated RNA processing and, to a lesser extent, intercellular RNA transfer [11,56,57]. In MPNs, the post-transcriptional processes can be further amplified in the inflamed BM microenvironment, where sustained exposure to inflammatory cytokines and chemokines promotes platelet priming and alters intracellular signaling pathways [11,49,58]. Through integration of clonal genetic programming and inflammatory stress, circulating platelets retain molecular signatures that capture key features of MPN disease biology.

A study employing RNA sequencing of platelets across all subtypes of MPNs demonstrated that platelet transcriptomes distinguish ET, PV, and MF and capture a continuum of disease progression towards MF [57]. Moreover, this study also showed that platelet RNA profiles appear responsive to JAK1/JAK2 inhibition, with ruxolitinib associated with suppression of inflammatory and interferon-related transcripts. These findings support platelet transcripts as a disease-responsive molecular readout in MPNs. Transcriptome-wide analyses provide an unbiased and comprehensive view of disease-associated transcriptional programs, which is essential for identifying novel disease-relevant pathways and candidate biomarkers. However, the resulting high-dimensional data require systematic reduction and validation to support clinical scalability.

An early platelet transcriptome sequencing study identified extensive transcriptional dysregulation in MF, with over 1000 genes uniquely altered relative to ET and PV [59]. From this high-dimensional dataset, a fibrosis-associated three-gene signature comprising *CEP55*, *CCND1*, and *H2AFX* was derived through statistical reduction and subsequently validated in an independent cohort, demonstrating its capacity to distinguish MPNs with fibrosis and without [59].

The three-gene fibrosis signature were statistically identified as best capturing MF [59]. Post hoc pathway analysis links them to genome stability and cell-cycle regulation [59]. A small number of studies have established the oncogenic relevance of these genes and demonstrated corresponding protein-level alterations and functional roles in megakaryocytes.

*CCND1* (Table 1) is a well-established oncogenic driver, exemplified in lymphoid malignancies where *CCND1* rearrangements define specific disease subtypes [60]. The *CCND1* transcript encodes cyclin D1, a key regulator of cell-cycle progression that facilitates polyploidization during megakaryocyte endomitosis, a process essential for normal megakaryocyte maturation [61]. Elevated cyclin D1 expression has further been linked to enhanced DNA replication and proliferative stress [61]. *H2AFX* (Table 1) encodes the histone variant *H2AX*, which upon phosphorylation at serine 139 forms γ-H2AX, a well-established marker of DNA double-strand breaks and genomic stress that coordinates DNA damage response signaling [62]. During megakaryocyte endomitosis, γ-H2AX facilitates recruitment of DNA repair proteins and supports genomic stability [63]. *CEP55* (Table 1), encoding the cytokinesis regulator centrosomal protein 55, is frequently overexpressed in human cancers [64,65]. Its dysregulation permits aberrant cell division and tolerates genomic instability, thereby driving tumorigenesis [64,65]. Given that megakaryocyte maturation depends on tightly regulated endomitosis and polyploidization, aberrant expression of these genes in platelets is likely to reflect upstream impairment of megakaryopoiesis. This may leave a transcriptional imprint that persists in circulating platelets and influences platelet biogenesis and function.

As these three fibrotic signature genes are primarily involved in genomic stability, their mechanistic relevance to platelet function remains to be elucidated. A recent study sought to explore the relationship between changes in platelet-associated genes and function using gene set enrichment analysis of gene expression data generated from peripheral blood. In silico analyses revealed that there were dysregulation of cytoskeletal and signaling-related genes in MPN that suggest there are defects in platelet activation, adhesion and immune regulation [44].

Achieving broad accessibility and reproducibility in platelet transcriptomics requires overcoming several important technical challenges. Pre-analytical and processing factors, such as sample collection, delays in processing, platelet isolation methods, and handling conditions, may all influence transcriptomic output and contribute to inter-study variability [65]. While some leukocyte-associated signals arise through biologically relevant processes such as emperipolesis, where exchange of genetic material between neutrophils and megakaryocytes has been demonstrated in platelets [67], transcripts derived from samples with contaminating leukocytes may also be falsely interpreted as platelet-associated signals. Given the low RNA content of platelets, even small amounts of leukocyte carryover can disproportionately influence bulk sequencing profiles, leading to substantial distortion of the apparent platelet transcriptome [68,69]. Recent spike-in studies systematically titrating leukocyte RNA into platelet preparations have helped define contamination thresholds compatible with robust platelet transcriptomic analysis, offering practical guidance for the field [17]. Continued methodological refinement and integration with orthogonal datasets will be essential to fully realize the potential of platelet transcriptomics.

### 5.2. Platelet Proteome

**Figure 2:**
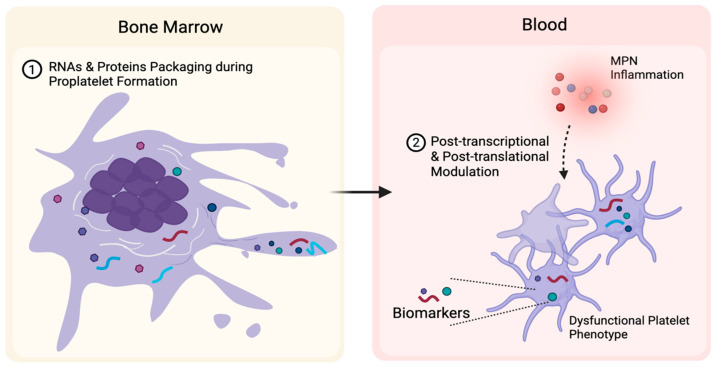
Platelets as carriers of MPN-specific signals and biomarkers. Platelets acquire MPN-associated molecular signatures by inheriting disease-imprinted RNAs and proteins from mutant megakaryocytes during proplatelet formation (**left**). After entering the circulation, platelet profiles are further reshaped through post-transcriptional and post-translational modulation in response to circulating microenvironmental signals, including the inflammatory milieu characteristic of MPNs (**right**). The altered molecular profile contributes to the platelet dysfunctional phenotype.

During thrombopoiesis, platelets inherit a repertoire of proteins from megakaryocytes, along with RNA molecular profiles (Figure 2) [15]. In parallel, megakaryocytes equip platelets with ribosomes, translation initiation factors, and spliceosomal components, which allows platelets to retain a limited capacity for post-transcriptional processing and stimulus-dependent protein synthesis from pre-existing mRNAs [15]. This confers platelets with a dynamic, adaptive capacity, enabling stimulus-dependent protein synthesis that modulates platelet functional responses following activation [70]. Such post-transcriptional and post-translational regulation gives rise to a pronounced transcript-protein gap, driven in part by alternative splicing and post-translational modifications (Figure 2) [16]. Therefore, direct interrogation of the platelet proteome is essential to capture disease-associated molecular alterations that cannot be inferred from transcriptomic analyses alone.

Advances in mass spectrometry-based technologies, including high-resolution, label-free Liquid Chromatography-Tandem Mass Spectrometry (LC-MS/MS) and data-independent acquisition such as Sequential Window Acquisition of all Theoretical Fragment Ion Spectra Mass Spectrometry (SWATH-MS), now enable increasingly sensitive and reproducible quantification of platelet proteins from limited clinical samples [71]. In MPNs, platelet proteomic profiling using LC-MS has identified substantial disease-associated alterations, including differential expression of multiple proteins in ET and PV compared with healthy controls [72]. The differences involve proteins linked to hypercoagulability, inflammatory signaling, and proteostasis, such as heat shock protein 47 (HSP47) and galectin-1 (Gal-1) [72]. The study identifies high-priority candidate proteins for future mechanistic investigation and highlights platelet proteomics as a valuable source of biomarkers in MPNs [72]. Furthermore, platelet proteome can provide a framework for subsequent investigation of platelet functional abnormalities underlying the hemostatic complications observed in MPNs.

Translation of platelet proteomic discoveries into clinical biomarkers will ultimately rely on targeted validation using orthogonal approaches such as immunoassays. Integration of platelet proteomics with transcriptomics, clinical phenotypes, and treatment exposure will be critical for prioritizing robust biomarker candidates and defining clinically meaningful protein signatures. Future studies incorporating longitudinal sampling and validation in independent cohorts will be essential to translate platelet proteomic discoveries into clinically applicable tools for disease monitoring, risk stratification, and assessment of treatment response in MPNs.

We focus here on several platelet-associated proteins identified across independent studies as being of biological relevance in MPNs. For each candidate, we evaluate their biological function, evidence of dysregulation in MPNs (Table 2), and their potential utility as dynamic biomarkers to address key clinical gaps.

AURKA

Aurora kinase A (AURKA) is a serine/threonine kinase essential for mitotic entry and progression, serving as a critical regulator of the G_2_/M phase transition [73]. Upregulation of AURKA has been documented in both platelets and megakaryocytes derived from MF patients, but the current functional evidence is limited to megakaryocytes [59,74]. The therapeutic potential of targeting AURKA was first demonstrated in preclinical studies, where MLN8237 (alisertib) induced megakaryocyte differentiation and polyploidization and reduced disease burden in *JAK2*^V617F^ and *MPL*^W515L^ mouse models of MF [74]. Clinical evaluation of alisertib in MF has since been undertaken in a Phase I study, which demonstrated preliminary safety and biologic activity [75]. These findings support AURKA as a promising therapeutic target in MF, particularly through its effects on abnormal megakaryopoiesis and disease burden. However, it remains unclear whether AURKA upregulation in platelets reflects a biologically active contributor to platelet phenotype.

Galectin-1

Gal-1 is a promising candidate protein due to its well-established roles in MPN pathophysiology. As a major platelet reservoir protein, Gal-1 is released upon platelet activation and in turn promote platelet aggregation, integrin αIIbβ3 activation, and platelet–leukocyte interactions [76]. These processes contribute to a self-reinforcing cycle of platelet activation and inflammation that is observed across MPN subtypes [76]. Recent work has identified Gal-1 as a central mediator within the proinflammatory stem cell niche of MF, where it was shown to be upregulated and functionally involved in sustaining fibrosis and disease progression [77]. Taken together, these findings provide a rationale for further validation of platelet-derived Gal-1 as a biomarker associated with fibrotic progression and thrombotic risk in MPNs.

Heat Shock Proteins

HSPs have garnered significant interest as potential therapeutic targets in hematologic malignancies such as MPNs [78]. In the stressed and inflammatory microenvironment of MPNs, upregulated HSP emerges as a potential regulator of disease pathogenesis by mediating platelet function, maintaining oncogenic signals and facilitating stress adaptation [78].

HSP47 is a collagen-specific molecular chaperone that plays a central role in collagen biosynthesis [79]. In platelets, HSP47 has been identified as a regulator of collagen-dependent activation, facilitating platelet adhesion and aggregation, and signaling in response to exposed collagen at sites of vascular injury [79,80]. Platelet-specific deletion or inhibition of HSP47 selectively impairs collagen- and GPVI-dependent platelet activation [79]. Beyond platelet function, dysregulated HSP47 expression has been implicated in fibrotic disorders, where it promotes collagen deposition [81]. In the context of MPNs, enhanced HSP47 activity may promote platelet hyperreactivity and thrombo-inflammatory signaling in environments of excessive extracellular matrix remodeling, with potential implications for fibrotic progression.

In platelets, HSP27 functions as a stress-inducible molecular chaperone that stabilizes cytoskeletal dynamics and is upregulated and phosphorylated in response to activation and stress, with phosphorylation correlating with enhanced platelet activation during agonist stimulation [82,83]. HSP27 has also been shown to stabilize the JAK2/STAT5 complex, enhancing STAT5 phosphorylation and sustaining its activation [84]. Sustained STAT5 activation may contribute to platelet priming downstream of TPO/MPL/JAK2 signaling. In platelets, TPO induces STAT5 phosphorylation and enhances responses to subthreshold agonists, including increased Ca^2+^ mobilization, secretion, and aggregation, while also supporting PI3K/Akt and Rap1B-associated αIIbβ3 activation [85,86,87]. In preclinical MPN models, HSP27 inhibition has been shown to attenuate fibrotic features, including reticulin deposition, megakaryocyte expansion, and platelet count abnormalities [78].

**Table 2:** Overview of platelet-associated proteins of interest in MPNs.

	Key Biological Function	Pathological Relevance to MPNs	Ref.
AURKA	Controls G_2_/M transition; suppresses megakaryocyte differentiation	Promotes immature platelet output; upregulated in MF, and inhibition reduces disease burden	[59,73,74]
Gal-1	Platelet aggregation, integrin αIIbβ3 activation, platelet–leukocyte interactions	Contributes to prothrombotic platelet behavior, inflammatory signaling, and fibrosis	[76,77]
HSP47	Supports collagen-dependent platelet activation and collagen deposition	Linked to enhanced platelet activation, thrombosis, and fibrotic progression	[79,80,81]
HSP27	Stress-response chaperone supporting platelet activation under inflammatory conditions	Stabilizes JAK2/STAT5 signaling; Inhibition reduces fibrosis	[78,82,83,84]

Current evidence suggests that HSP47 and HSP27 may link platelet activation, stress signaling, and fibrotic remodeling in MPNs, but their platelet-intrinsic mechanisms relevance and clinical significance remain to be demonstrated.

## 6. Conclusions

In conclusion, peripherally circulating platelets in MPNs provide a uniquely accessible molecular window into disease biology and risk. The study of platelets offers a promising avenue to elucidate MPN pathophysiology, and further research into their clinical applications is warranted, as ongoing advancements address current technical barriers. Characterizing platelet molecular and functional signatures holds promise for the development of novel biomarkers and for improving disease management across diagnostic, prognostic, and therapeutic contexts.

## Figures and Tables

**Table 1:** Overview of platelet-associated transcriptomes of interest in MPNs.

Gene	Key Biological Function	Role in Megakaryocytes	Dysregulation Implication in Platelets (MPNs)	Ref.
*CCND1*	Cell-cycle regulator	Facilitates polyploidization during megakaryocyte endomitosis	Impaired megakaryopoiesis link to altered platelet phenotypes	[60,61]
*H2AFX*	DNA damage marker	Recruits DNA repair proteins and promotes genomic stability during endomitosis		[62,66]
*CEP55*	Cytokinesis regulator	Regulates division-cycle		[64,65]

## Data Availability

No new data were created or analyzed in this study.

## Abbreviations

The following abbreviations are used in this manuscript:

AML	Acute myeloid leukemia
AURKA	Aurora kinase A
AvWS	Acquired von Willebrand syndrome
BM	Bone marrow
ERK 1/2	Extracellular signal-regulated kinase 1/2
ET	Essential thrombocythemia
Gal-1	Galectin-1
HSP47	Heat shock protein 47
IPSET	International Prognostic Score for Thrombosis in Essential Thrombocythemia
LC–MS/MS	Liquid chromatography–tandem mass spectrometry
MAPK	Mitogen-activated protein kinase
MF	Myelofibrosis
MPNs	Myeloproliferative neoplasms
PI3K	Phosphoinositide 3-kinase
PMF	Primary myelofibrosis
PV	Polycythemia vera
SWATH-MS	Sequential Window Acquisition of All Theoretical Fragment Ion Spectra Mass Spectrometry
TPO	Thrombopoietin
